# A two-dimensional manganese(II) complex: poly[bis­(μ_2_-4,4′-bipyrid­yl)tetra­kis­(μ_2_-3,5-dinitro­benzoato)dimanganese(II)]

**DOI:** 10.1107/S1600536810034495

**Published:** 2010-09-04

**Authors:** Jian Wang, Zhu-Lai Li, Xiu-Zhi Xu, Wen-Jin Yan

**Affiliations:** a Department of Medicinal Chemistry, School of Pharmacy, Fujian Medical University, Fuzhou, Fujian 350004, People’s Republic of China

## Abstract

The Mn atom in the title compound, [Mn_2_(C_7_H_3_N_2_O_6_)_4_(C_10_H_8_N_2_)_2_]_*n*_, is six-coordinated by two N atoms and four O atoms, forming a distorted octa­hedral geometry. The Mn—O bond lengths are in the range 2.1281 (13)–2.2011 (12) Å and the Mn—N bond lengths are 2.269 (2) and 2.278 (2) Å. Mn(II) atoms are double-bridged along the *a* axis by two pairs of bi-monodentate carboxyl groups, forming a double-stranded chain, while the bidentate 4,4′-bipyridine ligand bridges the Mn atom along the *b* axis. This results in a two-dimensional structure constructed of oblong grids with the sides of length 11.634 and 5.075 Å

## Related literature

In order to study the relationship between the manganese ion and the biological coordination agent, the role of the manganese ion in the active sites and the structure of the active sites in the manganese enzymes, small mol­ecule complexes are often applied to modeling the structure and the properties of reaction in the active centers, see: Shi *et al.* (2000 ▶). The characterization of metal complexes containing monocarb­oxy­lic acids has demonstrated the versatility of the carboxyl­ate group as an innersphere ligand, see: Mehrotra & Bohra (1983 ▶). 
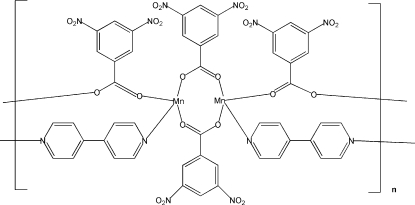


## Experimental

### 

#### Crystal data

[Mn_2_(C_7_H_3_N_2_O_6_)_4_(C_10_H_8_N_2_)_2_]
*M* *_r_* = 633.35
Monoclinic, 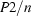

*a* = 10.0873 (6) Å
*b* = 11.6336 (6) Å
*c* = 21.2191 (12) Å
β = 98.075 (3)°
*V* = 2465.41 (14) Å^3^
*Z* = 4
Mo *K*α radiation
μ = 0.62 mm^−1^
*T* = 293 K
0.82 × 0.45 × 0.35 mm

#### Data collection

Rigaku Mercury diffractometer
Absorption correction: multi-scan (*CrystalStructure*; Rigaku, 2000 ▶) *T*_min_ = 0.779, *T*_max_ = 1.000
14626 measured reflections
4334 independent reflections
4149 reflections with *I* > 2σ(*I*)
*R*_int_ = 0.018

#### Refinement

*R*[*F* ^2^ > 2σ(*F* ^2^)] = 0.032
*wR*(*F* ^2^) = 0.085
*S* = 1.01
4334 reflections
393 parameters
H-atom parameters constrained
Δρ_max_ = 0.31 e Å^−3^
Δρ_min_ = −0.37 e Å^−3^

### 

Data collection: *CrystalClear* Rigaku (2000 ▶); cell refinement: *CrystalClear*; data reduction: *CrystalClear*; program(s) used to solve structure: *SHELXS97* (Sheldrick, 2008 ▶); program(s) used to refine structure: *SHELXL97* (Sheldrick, 2008 ▶); molecular graphics: *SHELXTL* (Sheldrick, 2008 ▶); software used to prepare material for publication: *SHELXL97*.

## Supplementary Material

Crystal structure: contains datablocks I, global. DOI: 10.1107/S1600536810034495/br2146sup1.cif

Structure factors: contains datablocks I. DOI: 10.1107/S1600536810034495/br2146Isup2.hkl

Additional supplementary materials:  crystallographic information; 3D view; checkCIF report

## Figures and Tables

**Fig. 1.**
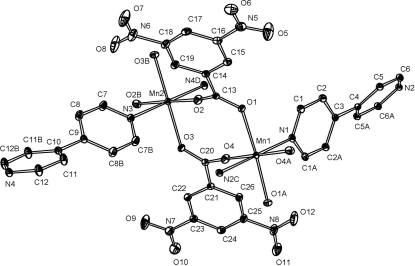
A view of the structure of the title compound, showing 30% probability displacement ellipsoids. H atoms have been omitted for clarity. Symmetry codes: (A) -x+1/2, y, -z+1/2; (B) -x+3/2, y, -z+1/2; (C) x, y+1, z;(D) x, y-1, z.

**Fig. 2.**
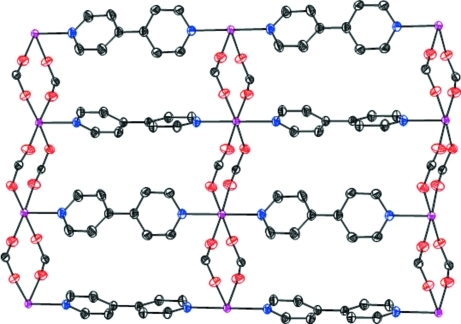
The two-dimensional structure of the title compound, constructed of oblong grids. Only the carboxyl group of the benzoic acid is shown for clarity.

## supplementary crystallographic information

### Comment

In order to study the relationship between the manganese ion and the biological coordination agent, the role of manganese ion in the active sites and the structure of the active sites in the manganese enzymes, small molecule complexes are often applied to modeling the structure and the properties of reaction in the active centers (Shi *et al.*2000). Metal complexes containing monocarboxylic acids are well known and the publication of many structurally characterized examples of this class of compounds has demonstrated the versatility of the carboxylate group as an innersphere ligand (Mehrotra *et al.*, 1983). In this paper, we will report the synthesis and crystal structure of a new two-dimensional manganese complex, [Mn_2_.(dinitrobenzoic acid)_4_.(4,4-bipy)_2_]n,(I). The crystal structure was confirmeded by X-ray crystallography.

The crystal structure of the title compound is illustrated in Fig.1. The Mn atom is located in an octahedral enviroment containing two N from two bipy ligand, four O from two chelating carboxyl. The Mn—O bond lengths are in the range 2.1281 (13) - 2.2011 (12) Å, and the Mn—N bond lengths are 2.269 (2) and 2.278 (2) Å.

Along the *a* axis, each Mn(II) center is double-bridged by two pair of bi-monodentate carboxyl, and forming a double-stranded chain. Along the *b* axis, the bidentate ligand 4,4'-bipyridine bridge the Mn atom in a straight mode. As a result, the whole molecule features a two-dimensional structure constructed of oblong grids with the side length 11.634 and 5.075 Å, as shown in Fig.2.

### Experimental

The reaction of dinitrobenzoic acid 0.636 g(3 mmol) and NaOH 0.12 g(3 mmol) in the molar ratio of 1:1 in an aqueous-alcohol(3:1) solution(40 ml) at room temperature for 30 minutes produced a colorless solution, to which MnCl_2_.6H_2_O 0.198 g(1 mmol) and 4,4'-bipyridine 0.156 g(1 mmol) was added to produced a yellowy solution. The reaction solution was kept at room temperature after stirring for an hour. Yellowy crystals were obtained after a few days, which were washed with water and ethanol for several times and then dried in air.

### Crystal data

**Table:**

[Mn_2_(C_7_H_3_N_2_O_6_)_4_(C_10_H_8_N_2_)_2_]	*F*(000) = 1284
*M**_r_* = 633.35	*D*_x_ = 1.706 Mg m^−^^3^
Monoclinic, *P*2/*n*	Mo *K*α radiation, λ = 0.71073 Å
Hall symbol: -P 2yac	Cell parameters from 5503 reflections
*a* = 10.0873 (6) Å	θ = 1.9–25.0°
*b* = 11.6336 (6) Å	µ = 0.62 mm^−^^1^
*c* = 21.2191 (12) Å	*T* = 293 K
β = 98.075 (3)°	Prism, yellow
*V* = 2465.41 (14) Å^3^	0.82 × 0.45 × 0.35 mm
*Z* = 4	

### Data collection

**Table:**

Rigaku Mercury diffractometer	4334 independent reflections
Radiation source: fine-focus sealed tube	4149 reflections with *I* > 2σ(*I*)
graphite	*R*_int_ = 0.018
ω scans	θ_max_ = 25.0°, θ_min_ = 2.0°
Absorption correction: multi-scan (*CrystalClear*; Rigaku, 2000)	*h* = −12→9
*T*_min_ = 0.779, *T*_max_ = 1.000	*k* = −13→13
14626 measured reflections	*l* = −23→25

### Refinement

**Table:**

Refinement on *F*^2^	Primary atom site location: structure-invariant direct methods
Least-squares matrix: full	Secondary atom site location: difference Fourier map
*R*[*F*^2^ > 2σ(*F*^2^)] = 0.032	Hydrogen site location: inferred from neighbouring sites
*wR*(*F*^2^) = 0.085	H-atom parameters constrained
*S* = 1.01	*w* = 1/[σ^2^(*F*_o_^2^) + (0.0435*P*)^2^ + 1.780*P*] where *P* = (*F*_o_^2^ + 2*F*_c_^2^)/3
4334 reflections	(Δ/σ)_max_ < 0.001
393 parameters	Δρ_max_ = 0.31 e Å^−^^3^
0 restraints	Δρ_min_ = −0.37 e Å^−^^3^

### Special details

**Table:**

Geometry. All e.s.d.'s (except the e.s.d. in the dihedral angle between two l.s. planes) are estimated using the full covariance matrix. The cell e.s.d.'s are taken into account individually in the estimation of e.s.d.'s in distances, angles and torsion angles; correlations between e.s.d.'s in cell parameters are only used when they are defined by crystal symmetry. An approximate (isotropic) treatment of cell e.s.d.'s is used for estimating e.s.d.'s involving l.s. planes.
Refinement. Refinement of *F*^2^ against ALL reflections. The weighted *R*-factor *wR* and goodness of fit *S* are based on *F*^2^, conventional *R*-factors *R* are based on *F*, with *F* set to zero for negative *F*^2^. The threshold expression of *F*^2^ > σ(*F*^2^) is used only for calculating *R*-factors(gt) *etc*. and is not relevant to the choice of reflections for refinement. *R*-factors based on *F*^2^ are statistically about twice as large as those based on *F*, and *R*- factors based on ALL data will be even larger.

### Fractional atomic coordinates and isotropic or equivalent isotropic displacement parameters (Å^2^)

**Table:**

	*x*	*y*	*z*	*U*_iso_*/*U*_eq_	
Mn1	0.2500	0.19545 (3)	0.2500	0.01900 (11)	
Mn2	0.7500	0.24424 (3)	0.2500	0.02079 (11)	
O1	0.39680 (12)	0.20294 (11)	0.33670 (6)	0.0285 (3)	
O2	0.59377 (14)	0.24937 (13)	0.30771 (7)	0.0376 (3)	
O3	0.60065 (13)	0.24451 (12)	0.16375 (6)	0.0317 (3)	
C13	0.51107 (17)	0.24788 (14)	0.34545 (8)	0.0228 (4)	
N4	0.7500	1.04904 (18)	0.2500	0.0289 (5)	
O4	0.39999 (13)	0.19047 (13)	0.18644 (6)	0.0378 (3)	
C3	0.2500	−0.2403 (2)	0.2500	0.0233 (5)	
C9	0.7500	0.6807 (2)	0.2500	0.0266 (5)	
N3	0.7500	0.43988 (18)	0.2500	0.0292 (5)	
C10	0.7500	0.8084 (2)	0.2500	0.0265 (5)	
N1	0.2500	0.00037 (17)	0.2500	0.0256 (4)	
O11	0.1529 (2)	−0.04745 (19)	−0.06614 (9)	0.0772 (7)	
C15	0.48226 (19)	0.28563 (16)	0.45950 (9)	0.0295 (4)	
H15	0.4092	0.2361	0.4548	0.035*	
N8	0.1882 (2)	−0.02270 (18)	−0.01070 (9)	0.0527 (5)	
C26	0.33672 (19)	0.09265 (16)	0.06677 (9)	0.0302 (4)	
H26	0.2875	0.0686	0.0983	0.036*	
C2	0.30203 (19)	−0.17792 (15)	0.30368 (9)	0.0294 (4)	
H2	0.3357	−0.2158	0.3411	0.035*	
C21	0.44557 (17)	0.16499 (15)	0.08170 (8)	0.0237 (4)	
N2	0.2500	−0.60873 (17)	0.2500	0.0247 (4)	
C14	0.55181 (18)	0.30668 (15)	0.40903 (8)	0.0255 (4)	
C4	0.2500	−0.3681 (2)	0.2500	0.0235 (5)	
N5	0.4524 (2)	0.31431 (18)	0.57173 (8)	0.0447 (5)	
C1	0.30335 (19)	−0.05938 (15)	0.30107 (9)	0.0290 (4)	
H1	0.3433	−0.0192	0.3366	0.035*	
O5	0.3718 (3)	0.2385 (3)	0.56666 (10)	0.1090 (11)	
C20	0.48563 (17)	0.20357 (14)	0.15039 (8)	0.0228 (4)	
C19	0.66089 (18)	0.38110 (16)	0.41675 (9)	0.0315 (4)	
H19	0.7101	0.3942	0.3835	0.038*	
C11	0.6523 (2)	0.87052 (17)	0.27427 (11)	0.0407 (5)	
H11	0.5844	0.8325	0.2913	0.049*	
C6	0.22428 (19)	−0.54874 (15)	0.30084 (8)	0.0276 (4)	
H6	0.2067	−0.5890	0.3366	0.033*	
C8	0.8668 (2)	0.61846 (17)	0.26293 (12)	0.0434 (5)	
H8	0.9485	0.6563	0.2718	0.052*	
C16	0.52304 (19)	0.33937 (17)	0.51722 (9)	0.0318 (4)	
O12	0.1347 (2)	−0.0599 (2)	0.03319 (10)	0.0937 (9)	
C5	0.22266 (18)	−0.42997 (15)	0.30270 (8)	0.0269 (4)	
H5	0.2035	−0.3918	0.3389	0.032*	
C17	0.6271 (2)	0.41653 (17)	0.52608 (9)	0.0345 (4)	
H17	0.6507	0.4542	0.5647	0.041*	
O10	0.5122 (2)	0.1732 (2)	−0.13269 (8)	0.0763 (7)	
O6	0.48179 (19)	0.36920 (17)	0.62041 (7)	0.0610 (5)	
N7	0.54582 (19)	0.20781 (19)	−0.07935 (8)	0.0457 (5)	
C7	0.8626 (2)	0.49998 (17)	0.26272 (12)	0.0436 (5)	
H7	0.9428	0.4600	0.2720	0.052*	
C22	0.51562 (18)	0.20179 (16)	0.03395 (9)	0.0288 (4)	
H22	0.5893	0.2500	0.0432	0.035*	
C23	0.47448 (19)	0.16581 (17)	−0.02799 (8)	0.0307 (4)	
C24	0.36823 (19)	0.09225 (17)	−0.04444 (9)	0.0326 (4)	
H24	0.3428	0.0682	−0.0862	0.039*	
O9	0.6350 (2)	0.2753 (3)	−0.06612 (9)	0.0986 (9)	
C12	0.6554 (2)	0.98865 (17)	0.27332 (11)	0.0409 (5)	
H12	0.5881	1.0285	0.2898	0.049*	
N6	0.8062 (2)	0.51773 (19)	0.48328 (11)	0.0562 (6)	
C25	0.30197 (19)	0.05647 (17)	0.00456 (9)	0.0326 (4)	
C18	0.69470 (19)	0.43534 (17)	0.47508 (10)	0.0356 (5)	
O7	0.8276 (2)	0.5696 (2)	0.53379 (11)	0.0904 (8)	
O8	0.8707 (2)	0.5303 (2)	0.43984 (12)	0.0902 (8)	

### Atomic displacement parameters (Å^2^)

**Table:**

	*U*^11^	*U*^22^	*U*^33^	*U*^12^	*U*^13^	*U*^23^
Mn1	0.0216 (2)	0.01424 (19)	0.02138 (19)	0.000	0.00381 (14)	0.000
Mn2	0.0215 (2)	0.01626 (19)	0.0247 (2)	0.000	0.00365 (14)	0.000
O1	0.0259 (7)	0.0332 (7)	0.0253 (6)	−0.0066 (5)	0.0001 (5)	0.0004 (5)
O2	0.0320 (7)	0.0491 (9)	0.0339 (7)	−0.0046 (6)	0.0124 (6)	−0.0083 (6)
O3	0.0273 (7)	0.0408 (8)	0.0261 (7)	−0.0089 (6)	0.0006 (5)	0.0002 (6)
C13	0.0231 (9)	0.0201 (8)	0.0246 (9)	0.0025 (7)	0.0009 (7)	0.0027 (7)
N4	0.0333 (12)	0.0192 (10)	0.0350 (12)	0.000	0.0079 (9)	0.000
O4	0.0292 (7)	0.0581 (9)	0.0283 (7)	−0.0048 (6)	0.0117 (6)	−0.0079 (6)
C3	0.0243 (13)	0.0157 (12)	0.0301 (13)	0.000	0.0041 (10)	0.000
C9	0.0300 (13)	0.0201 (12)	0.0301 (13)	0.000	0.0059 (11)	0.000
N3	0.0265 (11)	0.0195 (10)	0.0409 (13)	0.000	0.0028 (9)	0.000
C10	0.0298 (13)	0.0194 (12)	0.0294 (13)	0.000	0.0009 (11)	0.000
N1	0.0272 (11)	0.0172 (10)	0.0329 (11)	0.000	0.0061 (9)	0.000
O11	0.0918 (15)	0.0888 (15)	0.0484 (11)	−0.0533 (12)	0.0010 (10)	−0.0245 (10)
C15	0.0276 (9)	0.0302 (9)	0.0296 (10)	0.0000 (8)	0.0004 (8)	−0.0021 (8)
N8	0.0582 (13)	0.0540 (12)	0.0446 (11)	−0.0287 (10)	0.0026 (9)	−0.0110 (9)
C26	0.0309 (10)	0.0306 (10)	0.0297 (10)	−0.0051 (8)	0.0065 (8)	−0.0023 (8)
C2	0.0355 (10)	0.0211 (9)	0.0300 (10)	0.0026 (8)	−0.0003 (8)	0.0011 (7)
C21	0.0229 (8)	0.0238 (8)	0.0244 (9)	0.0023 (7)	0.0031 (7)	−0.0018 (7)
N2	0.0307 (11)	0.0168 (10)	0.0261 (11)	0.000	0.0021 (9)	0.000
C14	0.0241 (9)	0.0228 (9)	0.0283 (9)	0.0039 (7)	−0.0006 (7)	−0.0017 (7)
C4	0.0230 (12)	0.0170 (12)	0.0294 (13)	0.000	−0.0001 (10)	0.000
N5	0.0488 (11)	0.0559 (12)	0.0288 (9)	0.0035 (10)	0.0029 (8)	−0.0019 (8)
C1	0.0344 (10)	0.0207 (9)	0.0314 (10)	0.0010 (8)	0.0032 (8)	−0.0046 (7)
O5	0.129 (2)	0.155 (2)	0.0501 (12)	−0.092 (2)	0.0402 (13)	−0.0271 (14)
C20	0.0235 (9)	0.0208 (8)	0.0242 (9)	0.0028 (7)	0.0037 (7)	−0.0009 (7)
C19	0.0258 (9)	0.0296 (10)	0.0387 (10)	0.0010 (8)	0.0029 (8)	−0.0025 (8)
C11	0.0405 (11)	0.0227 (10)	0.0640 (14)	−0.0020 (9)	0.0255 (10)	0.0008 (9)
C6	0.0373 (10)	0.0213 (9)	0.0244 (9)	−0.0011 (7)	0.0053 (7)	0.0016 (7)
C8	0.0258 (10)	0.0238 (10)	0.0782 (16)	−0.0040 (8)	−0.0008 (10)	−0.0016 (10)
C16	0.0331 (10)	0.0334 (10)	0.0279 (10)	0.0075 (8)	0.0006 (8)	−0.0004 (8)
O12	0.1066 (17)	0.1188 (19)	0.0565 (12)	−0.0850 (16)	0.0147 (11)	−0.0096 (12)
C5	0.0331 (10)	0.0217 (9)	0.0259 (9)	0.0005 (7)	0.0041 (7)	−0.0034 (7)
C17	0.0354 (11)	0.0319 (10)	0.0329 (10)	0.0088 (8)	−0.0072 (8)	−0.0091 (8)
O10	0.0802 (13)	0.1256 (19)	0.0245 (9)	−0.0335 (13)	0.0120 (8)	−0.0062 (10)
O6	0.0786 (12)	0.0728 (12)	0.0314 (8)	0.0081 (10)	0.0075 (8)	−0.0120 (8)
N7	0.0422 (11)	0.0696 (13)	0.0261 (9)	−0.0103 (10)	0.0076 (8)	0.0012 (9)
C7	0.0258 (10)	0.0231 (10)	0.0790 (16)	0.0016 (8)	−0.0033 (10)	−0.0001 (10)
C22	0.0249 (9)	0.0332 (10)	0.0280 (9)	−0.0032 (7)	0.0029 (7)	−0.0015 (7)
C23	0.0305 (10)	0.0385 (10)	0.0236 (9)	0.0000 (8)	0.0056 (7)	0.0013 (8)
C24	0.0359 (10)	0.0350 (10)	0.0254 (9)	0.0022 (8)	−0.0005 (8)	−0.0059 (8)
O9	0.0972 (17)	0.160 (2)	0.0429 (11)	−0.0870 (17)	0.0259 (11)	−0.0133 (12)
C12	0.0427 (12)	0.0220 (10)	0.0631 (14)	0.0029 (9)	0.0251 (10)	−0.0008 (9)
N6	0.0444 (11)	0.0505 (12)	0.0721 (15)	−0.0161 (10)	0.0029 (11)	−0.0210 (11)
C25	0.0321 (10)	0.0300 (10)	0.0347 (10)	−0.0075 (8)	0.0018 (8)	−0.0055 (8)
C18	0.0273 (10)	0.0277 (10)	0.0487 (12)	−0.0003 (8)	−0.0053 (9)	−0.0079 (9)
O7	0.0861 (15)	0.0873 (16)	0.0976 (17)	−0.0454 (13)	0.0123 (13)	−0.0546 (14)
O8	0.0725 (14)	0.1070 (18)	0.0951 (16)	−0.0562 (13)	0.0260 (12)	−0.0321 (14)

### Geometric parameters (Å, °)

**Table:**

Mn1—O4^i^	2.1644 (13)	C26—H26	0.9300
Mn1—O4	2.1644 (13)	C2—C1	1.380 (3)
Mn1—O1	2.1954 (12)	C2—H2	0.9300
Mn1—O1^i^	2.1954 (12)	C21—C22	1.382 (3)
Mn1—N1	2.269 (2)	C21—C20	1.524 (2)
Mn1—N2^ii^	2.278 (2)	N2—C6	1.340 (2)
Mn2—O2^iii^	2.1281 (13)	N2—C6^i^	1.340 (2)
Mn2—O2	2.1281 (13)	N2—Mn1^iv^	2.278 (2)
Mn2—O3	2.2011 (12)	C14—C19	1.391 (3)
Mn2—O3^iii^	2.2011 (12)	C4—C5^i^	1.390 (2)
Mn2—N4^iv^	2.271 (2)	C4—C5	1.390 (2)
Mn2—N3	2.276 (2)	N5—O5	1.194 (3)
O1—C13	1.255 (2)	N5—O6	1.215 (2)
O2—C13	1.235 (2)	N5—C16	1.470 (3)
O3—C20	1.249 (2)	C1—H1	0.9300
C13—C14	1.517 (2)	C19—C18	1.388 (3)
N4—C12	1.335 (2)	C19—H19	0.9300
N4—C12^iii^	1.335 (2)	C11—C12	1.375 (3)
N4—Mn2^ii^	2.271 (2)	C11—H11	0.9300
O4—C20	1.241 (2)	C6—C5	1.382 (3)
C3—C2	1.390 (2)	C6—H6	0.9300
C3—C2^i^	1.390 (2)	C8—C7	1.379 (3)
C3—C4	1.487 (3)	C8—H8	0.9300
C9—C8^iii^	1.377 (2)	C16—C17	1.374 (3)
C9—C8	1.377 (2)	C5—H5	0.9300
C9—C10	1.485 (3)	C17—C18	1.375 (3)
N3—C7	1.329 (2)	C17—H17	0.9300
N3—C7^iii^	1.329 (2)	O10—N7	1.205 (2)
C10—C11^iii^	1.379 (2)	N7—O9	1.198 (3)
C10—C11	1.379 (2)	N7—C23	1.471 (2)
N1—C1^i^	1.335 (2)	C7—H7	0.9300
N1—C1	1.335 (2)	C22—C23	1.386 (3)
O11—N8	1.215 (3)	C22—H22	0.9300
C15—C14	1.382 (3)	C23—C24	1.378 (3)
C15—C16	1.385 (3)	C24—C25	1.377 (3)
C15—H15	0.9300	C24—H24	0.9300
N8—O12	1.219 (3)	C12—H12	0.9300
N8—C25	1.472 (3)	N6—O8	1.209 (3)
C26—C25	1.383 (3)	N6—O7	1.223 (3)
C26—C21	1.384 (3)	N6—C18	1.470 (3)
			
O4^i^—Mn1—O4	176.93 (8)	C22—C21—C20	121.03 (16)
O4^i^—Mn1—O1	85.82 (5)	C26—C21—C20	119.53 (16)
O4—Mn1—O1	94.30 (5)	C6—N2—C6^i^	117.2 (2)
O4^i^—Mn1—O1^i^	94.30 (5)	C6—N2—Mn1^iv^	121.38 (10)
O4—Mn1—O1^i^	85.82 (5)	C6^i^—N2—Mn1^iv^	121.38 (10)
O1—Mn1—O1^i^	175.45 (7)	C15—C14—C19	119.86 (17)
O4^i^—Mn1—N1	88.47 (4)	C15—C14—C13	120.37 (16)
O4—Mn1—N1	88.47 (4)	C19—C14—C13	119.76 (16)
O1—Mn1—N1	92.27 (3)	C5^i^—C4—C5	117.6 (2)
O1^i^—Mn1—N1	92.27 (3)	C5^i^—C4—C3	121.18 (11)
O4^i^—Mn1—N2^ii^	91.53 (4)	C5—C4—C3	121.18 (11)
O4—Mn1—N2^ii^	91.53 (4)	O5—N5—O6	123.1 (2)
O1—Mn1—N2^ii^	87.73 (3)	O5—N5—C16	118.33 (18)
O1^i^—Mn1—N2^ii^	87.73 (3)	O6—N5—C16	118.5 (2)
N1—Mn1—N2^ii^	180.0	N1—C1—C2	123.20 (17)
O2^iii^—Mn2—O2	176.79 (8)	N1—C1—H1	118.4
O2^iii^—Mn2—O3	89.84 (5)	C2—C1—H1	118.4
O2—Mn2—O3	90.16 (5)	O4—C20—O3	127.70 (17)
O2^iii^—Mn2—O3^iii^	90.16 (5)	O4—C20—C21	116.05 (15)
O2—Mn2—O3^iii^	89.84 (5)	O3—C20—C21	116.25 (15)
O3—Mn2—O3^iii^	179.83 (7)	C18—C19—C14	118.62 (18)
O2^iii^—Mn2—N4^iv^	91.61 (4)	C18—C19—H19	120.7
O2—Mn2—N4^iv^	91.61 (4)	C14—C19—H19	120.7
O3—Mn2—N4^iv^	90.08 (4)	C12—C11—C10	120.00 (19)
O3^iii^—Mn2—N4^iv^	90.08 (4)	C12—C11—H11	120.0
O2^iii^—Mn2—N3	88.39 (4)	C10—C11—H11	120.0
O2—Mn2—N3	88.39 (4)	N2—C6—C5	123.22 (17)
O3—Mn2—N3	89.92 (4)	N2—C6—H6	118.4
O3^iii^—Mn2—N3	89.92 (4)	C5—C6—H6	118.4
N4^iv^—Mn2—N3	180.0	C9—C8—C7	120.00 (19)
C13—O1—Mn1	129.61 (11)	C9—C8—H8	120.0
C13—O2—Mn2	174.27 (14)	C7—C8—H8	120.0
C20—O3—Mn2	133.90 (12)	C17—C16—C15	122.84 (18)
O2—C13—O1	126.85 (16)	C17—C16—N5	117.85 (17)
O2—C13—C14	116.55 (16)	C15—C16—N5	119.31 (18)
O1—C13—C14	116.60 (15)	C6—C5—C4	119.34 (17)
C12—N4—C12^iii^	116.5 (2)	C6—C5—H5	120.3
C12—N4—Mn2^ii^	121.75 (11)	C4—C5—H5	120.3
C12^iii^—N4—Mn2^ii^	121.75 (11)	C16—C17—C18	116.74 (17)
C20—O4—Mn1	171.41 (14)	C16—C17—H17	121.6
C2—C3—C2^i^	117.1 (2)	C18—C17—H17	121.6
C2—C3—C4	121.47 (11)	O9—N7—O10	122.9 (2)
C2^i^—C3—C4	121.47 (11)	O9—N7—C23	118.28 (18)
C8^iii^—C9—C8	116.5 (2)	O10—N7—C23	118.85 (19)
C8^iii^—C9—C10	121.73 (12)	N3—C7—C8	123.49 (19)
C8—C9—C10	121.73 (12)	N3—C7—H7	118.3
C7—N3—C7^iii^	116.5 (2)	C8—C7—H7	118.3
C7—N3—Mn2	121.75 (12)	C21—C22—C23	119.06 (17)
C7^iii^—N3—Mn2	121.75 (12)	C21—C22—H22	120.5
C11^iii^—C10—C11	116.8 (2)	C23—C22—H22	120.5
C11^iii^—C10—C9	121.62 (12)	C24—C23—C22	123.12 (17)
C11—C10—C9	121.62 (12)	C24—C23—N7	117.57 (17)
C1^i^—N1—C1	117.3 (2)	C22—C23—N7	119.31 (17)
C1^i^—N1—Mn1	121.37 (11)	C25—C24—C23	116.06 (17)
C1—N1—Mn1	121.37 (11)	C25—C24—H24	122.0
C14—C15—C16	119.02 (18)	C23—C24—H24	122.0
C14—C15—H15	120.5	N4—C12—C11	123.38 (19)
C16—C15—H15	120.5	N4—C12—H12	118.3
O11—N8—O12	123.7 (2)	C11—C12—H12	118.3
O11—N8—C25	118.4 (2)	O8—N6—O7	124.1 (2)
O12—N8—C25	117.96 (19)	O8—N6—C18	118.3 (2)
C25—C26—C21	119.33 (17)	O7—N6—C18	117.6 (2)
C25—C26—H26	120.3	C24—C25—C26	122.95 (18)
C21—C26—H26	120.3	C24—C25—N8	117.98 (18)
C1—C2—C3	119.56 (17)	C26—C25—N8	119.07 (18)
C1—C2—H2	120.2	C17—C18—C19	122.84 (18)
C3—C2—H2	120.2	C17—C18—N6	117.80 (18)
C22—C21—C26	119.44 (17)	C19—C18—N6	119.35 (19)
			
C4—C3—C2—C1	178.24 (13)	C10—C9—C8—C7	179.75 (18)
C2—C3—C4—C5	39.28 (13)	C8—C9—C10—C11^iii^	39.15 (17)
C2^i^—C3—C4—C5	−140.72 (13)	C8—C9—C10—C11	−140.85 (17)

Symmetry codes: (i) −*x*+1/2, *y*, −*z*+1/2; (ii) *x*, *y*+1, *z*; (iii) −*x*+3/2, *y*, −*z*+1/2; (iv) *x*, *y*−1, *z*.
